# Stage of Change, Self-Efficacy, and Barriers to Plant-Based Eating: A Secondary Analysis of Residential Program Data

**DOI:** 10.7759/cureus.111650

**Published:** 2026-06-28

**Authors:** David M Goldman, Matthew Nagra, Laurie Kortowich, Adam L Sud, Sachin Shah

**Affiliations:** 1 Research and Development, Metabite, New York, USA; 2 Public Health, University of Helsinki, Helsinki, FIN; 3 Family Practice, University of British Columbia, Vancouver, CAN; 4 Nutrition, Plant Strong, Austin, USA; 5 Nutrition, Adam Sud, LLC, Austin, USA; 6 Cardiology, Thomas J. Long School of Pharmacy, University of the Pacific, Stockton, USA

**Keywords:** behavior change, food and nutrition, nutritional intervention, plant-based diet, transtheoretical model

## Abstract

Introduction

Plant-based dietary patterns reduce cardiometabolic risk, yet sustained adoption remains limited. Psychosocial factors, including readiness for change and self-efficacy, are central to dietary behavior change, but their distribution among adults attending immersive lifestyle interventions has not been formally characterized. This study describes the stage of change, perceived barriers and facilitators, and self-efficacy among Plant Strong retreat attendees.

Materials and methods

Program evaluation surveys collected during routine operations were made available for secondary research analysis; 73 of 92 surveys were completed. Measures included the transtheoretical model (TTM) stage of change, self-reported dietary pattern, barriers and facilitators to whole-food, plant-based (WFPB) eating, and a two-item self-efficacy composite. One-way ANOVA with Tukey honestly significant difference (HSD) post-hoc analyses examined self-efficacy by TTM stage; chi-square tests assessed dietary pattern associations with barriers and facilitators.

Results

Nearly half (n = 35, 47.9%) of participants were in the maintenance stage. Self-efficacy was significantly higher among groups further along the TTM continuum (F(3, 69) = 7.34, p = 0.0002, η2 = 0.242), with the pre-action group scoring significantly lower than action (p = 0.013) or maintenance (p = 0.0002). Common barriers were food availability when eating out (n = 62, 84.9%) and meal preparation time (n = 54, 73.9%). Improving long-term health was universally endorsed as very helpful (n = 68, 93.2%). Dietary pattern showed nominally significant associations with barriers related to taste and texture, meal preparation knowledge, and preparation time (all p ≤ 0.032); none survived Benjamini-Hochberg correction across 12 simultaneous tests (false discovery rate-adjusted p ≥ 0.062).

Conclusions

Self-efficacy varied with TTM stage in a motivated retreat population, consistent with the dietary behavior change literature. The small pre-action subgroup and persistent barriers, particularly food availability away from home, suggest post-retreat support targeting eating-out contexts may strengthen sustained behavior change.

## Introduction

Cardiac, renal, and metabolic conditions, including cardiovascular diseases (CVD), type 2 diabetes (T2D), and chronic kidney disease, are leading causes of morbidity and mortality in the United States, with more than one in four adults affected by at least one of these conditions as of 2020, a figure that has risen steadily over the past two decades [1]. Dietary modification is among the major modifiable factors for reducing cardiometabolic risk [2]. Plant-based dietary patterns, in particular, are associated with reductions in total and LDL cholesterol, body weight, CVD risk, and all-cause mortality [3,4], with significant improvements observed even over short intervention windows [5-7]. Despite a growing evidence base, population-level adoption remains limited [8]. The gap between what people know about healthy eating and what they actually consume appears to be shaped less by information alone and more by psychosocial factors: habitual eating patterns, social pressure, limited cooking knowledge, cost, and low self-efficacy for sustaining change [9].

Theoretical models offer one system for understanding this gap. The transtheoretical model (TTM) categorizes individuals across stages of behavioral readiness (precontemplation, contemplation, preparation, action, and maintenance) and has been applied with some success to dietary behavior, including meat reduction [10,11]. Stage of change has been shown to correlate with dietary behavior and demonstrates a consistent positive relationship with self-efficacy [12], which is among the strongest psychosocial predictors of sustained dietary adherence and an important target for lifestyle medicine programs [13,14]. What remains less clear is how these constructs distribute among adults who voluntarily enroll in intensive, immersive lifestyle interventions, a population that likely differs meaningfully from the general public yet has received little focused empirical attention.

Intensive lifestyle programs incorporating plant-based dietary principles have demonstrated benefits in real-life settings. Comprehensive cardiac rehabilitation enhanced by a plant-based diet, for example, produced greater reductions in LDL cholesterol, body weight, and systolic blood pressure than standard rehabilitation alone [15]. The Plant Strong retreat program, a multi-day residential intervention incorporating medical oversight, evidence-based nutrition education, culinary instruction, and structured physical activity, operates within this tradition. Retrospective analyses of its biometric outcomes are underway; however, the psychosocial profile of its participants has not been formally characterized. This study aims to address that gap with two objectives: (1) to describe the distribution of the TTM stage of change, self-efficacy, and perceived barriers and facilitators to WFPB eating among adults attending a six-day residential Plant Strong retreat in April 2026, and (2) to examine whether self-efficacy differs by TTM stage and whether perceived barriers and facilitators differ by current dietary pattern. The results may inform program design and post-retreat support strategies that target sustained behavioral change.

## Materials and methods

Study design

This study presents an analysis of cross-sectional survey data assessing attitudes, behaviors, and psychosocial factors related to whole-food, plant-based (WFPB) eating among adults who attended a Plant Strong immersive health retreat. As part of routine program practice, data had been collected through a standardized program evaluation survey administered during routine retreat operations, and were subsequently made available for retrospective research analysis in accordance with an approved IRB protocol from the University of the Pacific (Protocol #IRB2026-128). All data were analyzed in aggregate without retention of individually identifiable information.

Setting and participants

The retreat was held April 19-24, 2026, at Blue Ridge Assembly in Black Mountain, North Carolina. The Plant Strong retreat is a six-day residential lifestyle intervention incorporating evidence-based educational sessions led by board-certified physicians, culinary instruction, daily physical activity, and community programming. All 92 adult attendees (minimum age 18) were eligible; 73 completed the survey, yielding a response rate of 79.3%. Because participation was voluntary and anonymous, nonrespondents could not be characterized, and findings should be interpreted as reflecting the responding subsample.

Survey instrument

The instrument was developed to assess psychosocial constructs relevant to WFPB dietary adoption and was optimized for mobile completion in approximately four to five minutes. Following an informed consent acknowledgment, five sections were administered. Demographics collected included age, gender identity, education level, healthcare provider status, ethnicity, and race; health status and medical comorbidities were not assessed. Dietary readiness was assessed with two complementary items: a TTM stage-of-change item categorizing participants from precontemplation through maintenance with respect to WFPB eating, and a behavioral item capturing self-reported dietary pattern over the preceding 30 days (omnivorous, plant-forward, vegetarian, or entirely plant-based). Both items were retained because motivational readiness and current behavior do not uniformly align, and each contributes independent information. Barriers (seven items, e.g., food availability, social pressure, cost) and facilitators (five items, e.g., health motivation, household support, recipe access) were each rated on three-point scales (not a barrier/factor → significant barrier/very helpful). Three-point formats were selected to minimize respondent burden in the time-limited retreat context. Self-efficacy was assessed with two five-point items (1 = not at all confident, 5 = completely confident) targeting confidence to maintain a WFPB diet under stress and during social events or holidays, two contexts consistently linked to dietary lapse. No validated WFPB-specific self-efficacy scale was available; this two-item operationalization is acknowledged as a study limitation. Similarly, the barrier and facilitator items were developed for this program evaluation and have not been independently validated.

Survey administration

The survey was distributed by email via Google Forms (Google LLC, Mountain View, CA) one day preceding the retreat, with reminders delivered by email and in-person announcements throughout the event. Surveys completed within 24 hours of the retreat's conclusion were included. As survey administration spanned the full retreat period, responses may reflect varying degrees of cumulative exposure to retreat programming depending on when during the retreat the survey was completed. No personally identifiable information was retained.

Statistical analysis

All analyses were conducted in IBM SPSS Statistics, Version 31 (IBM Corp., Armonk, NY), with a two-sided α = 0.05. Continuous variables are reported as means (SD); categorical variables as frequencies and percentages; barrier and facilitator items as frequency distributions. Healthcare provider status (n = 8, 11.0%) is reported descriptively only, as the subsample was insufficient for reliable inferential comparison. Self-efficacy items are reported individually and as a composite score, calculated as the mean of the two items for each participant; Cronbach’s α is reported to characterize item coherence. To examine self-efficacy by TTM stage, precontemplation and contemplation were combined a priori into a single “pre-action” category due to sparse cell sizes, yielding four analytic groups (pre-action, preparation, action, maintenance); all five stages are reported descriptively. A one-way ANOVA with Tukey honestly significant difference (HSD) post-hoc comparisons was conducted, with Kruskal-Wallis as a nonparametric sensitivity analysis. Dietary pattern associations with barrier and facilitator items were assessed via chi-square tests of independence (Fisher's exact where expected cells fell below five); effect sizes are reported as Cramér's V. All inferential findings are considered exploratory. No correction for multiple comparisons was applied to the 12 chi-square tests, given the exploratory nature of the analyses; false-discovery rate (FDR)-adjusted p-values (Benjamini-Hochberg correction across 12 simultaneous tests) are reported alongside raw p-values for transparency. No data were missing across any item.

## Results

Participants

Of the 92 eligible retreat attendees, 73 completed the survey (response rate = 79.3% (95% CI: 70.0-86.4%)). The sample was predominantly female (78.1% (67.3-86.0%), n = 57) and White (97.3% (90.5-99.2%), n = 71), with a mean age of 63.3 years (SD = 10.2; range: 36-82). Educational attainment was high: 43.8% (33.0-55.2%) (n = 32) held a master's degree, 28.8% (19.7-40.0%) (n = 21) a bachelor's degree, and 11.0% (5.7-20.2%) (n = 8) a doctorate or professional degree. Eight participants (11.0% (5.7-20.2%)) identified as healthcare providers; this subgroup is reported descriptively only.

Dietary stage of change and current dietary pattern

Nearly half the sample (47.9% (95% CI: 36.9-59.2%), n = 35) was in the maintenance stage, indicating sustained adherence to a WFPB diet for more than six months. An additional 26.0% (17.3-37.1%) (n = 19) were in the preparation stage, 15.1% (8.6-25.0%) (n = 11) in the action stage, 8.2% (3.8-16.8%) (n = 6) in the contemplation stage, and 2.7% (0.8-9.5%) (n = 2) in the pre-contemplation stage. Corresponding dietary patterns over the preceding 30 days were as follows: entirely plant-based, 47.9% (36.9-59.2%) (n = 35); plant-forward, 23.3% (15.1-34.2%) (n = 17); omnivorous, 17.8% (10.7-28.1%) (n = 13); and vegetarian, 11.0% (5.7-20.2%) (n = 8).

Perceived barriers

The most commonly reported barriers were food availability when eating out (84.9%, n = 62 rated as at least a minor barrier; 41.1%, n = 30 significant), time for meal preparation (73.9%, n = 54; 34.2%, n = 25 significant), and social pressure from friends or family (58.9%, n = 43; 21.9%, n = 16 significant). Knowledge for meal preparation was a minor or significant barrier for 49.3% (n = 36) of respondents. Cost (20.5%, n = 15) and conflicting nutritional information (35.6%, n = 26) were the least commonly reported barriers. Frequency distributions for all barrier and facilitator items are presented in Table 1 and Table 2.

**Table 1:** Perceived Barriers to Whole-Food, Plant-Based Eating (N = 73) Values are n (%). Barriers ordered by frequency of “Significant Barrier” response (descending).

Barrier Item	Not a Barrier % (n) (95% CI)	Minor Barrier % (n) (95% CI)	Significant Barrier % (n) (95% CI)
Food availability when eating out	15.1% (11) (8.6-25.0)	43.8% (32) (33.0-55.2)	41.1% (30) (30.5-52.6)
Time for meal preparation	26.0% (19) (17.3-37.1)	39.7% (29) (29.3-51.2)	34.2% (25) (24.4-45.7)
Social pressure from friends or family	41.1% (30) (30.5-52.6)	37.0% (27) (26.8-48.5)	21.9% (16) (14.0-32.7)
Knowledge for meal preparation	50.7% (37) (39.5-61.8)	34.2% (25) (24.4-45.7)	15.1% (11) (8.6-25.0)
Taste or texture of plant-based foods	65.8% (48) (54.3-75.6)	26.0% (19) (17.3-37.1)	8.2% (6) (3.8-16.8)
Conflicting nutritional information	64.4% (47) (52.9-74.4)	27.4% (20) (18.5-38.6)	8.2% (6) (3.8-16.8)
Cost	79.5% (58) (68.8-87.1)	19.2% (14) (11.8-29.7)	1.4% (1) (0.2-7.4)

**Table 2:** Perceived Facilitators to Whole-Food, Plant-Based Eating (N = 73) Values are n (%). Facilitators ordered by frequency of “Very Helpful” response (descending).

Facilitator Item	Not a Factor % (n) (95% CI)	Somewhat Helpful % (n) (95% CI)	Very Helpful % (n) (95% CI)
Desire to improve long-term health	1.4% (1) (0.2-7.4)	5.5% (4) (2.2-13.3)	93.2% (68) (84.9-97.0)
Physical energy gains	4.1% (3) (1.4-11.4)	13.7% (10) (7.6-23.4)	82.2% (60) (71.9-89.3)
Access to recipes or meal plans	8.2% (6) (3.8-16.8)	17.8% (13) (10.7-28.1)	74.0% (54) (62.9-82.7)
Household support	43.8% (32) (33.0-55.2)	12.3% (9) (6.6-21.8)	43.8% (32) (33.0-55.2)
Concern for animal welfare or the environment	17.8% (13) (10.7-28.1)	47.9% (35) (36.9-59.2)	34.2% (25) (24.4-45.7)

Perceived facilitators

Desire to improve long-term health was nearly universally reported as very helpful (93.2%, n = 68). Physical energy gains (82.2%, n = 60) and access to recipes or meal plans (74.0%, n = 54) were also highly rated. Household support was rated very helpful by 43.8% (n = 32) and not a factor by an equal proportion (43.8%, n = 32), reflecting marked between-person variability. Concern for animal welfare or the environment was reported as very helpful by 34.2% (n = 25) and as not a factor by 17.8% (n = 13).

Self-efficacy

Mean self-efficacy for maintaining a WFPB diet under stress was 3.82 (SD = 1.07; 95% CI: 3.57-4.07) and during social gatherings or holidays was 3.70 (SD = 1.09; 95% CI: 3.45-3.95), both on a five-point scale. The two-item composite score was 3.76 (SD = 0.95; median = 4.00; range: 1.00-5.00), indicating moderately high overall confidence. The two items demonstrated acceptable internal consistency (Cronbach’s α = 0.718). Self-efficacy scores by TTM stage are presented in Table 3.

**Table 3:** Composite Self-Efficacy Scores by TTM Stage of Change Self-efficacy evaluated on a five-point scale. SD: standard deviation; TTM: transtheoretical model

Stage of Change	n	Mean Self-Efficacy (SD)	95% Confidence Interval
Pre-action (contemplation/pre-contemplation)	8	2.62 (1.19)	(1.37, 3.87)
Preparation	19	3.53 (0.98)	(3.06, 4.00)
Action	11	3.86 (0.84)	(3.30, 4.42)
Maintenance	35	4.11 (0.68)	(3.88, 4.34)
Total sample	73	3.76 (0.95)	(3.54, 3.98)

Self-efficacy by TTM stage

A one-way ANOVA revealed a statistically significant difference in composite self-efficacy across the four TTM groups, F(3, 69) = 7.34, p = 0.0002, η² = 0.242, indicating a large effect. Group means were progressively higher across stages, from pre-action (M = 2.62, SD = 1.19) through preparation (M = 3.53, SD = 0.98), action (M = 3.86, SD = 0.84), to maintenance (M = 4.11, SD = 0.68). Tukey HSD post-hoc comparisons indicated that the pre-action group differed significantly from both the action group (p = 0.013) and the maintenance group (p = 0.0002). No other pairwise comparisons reached significance (all p-values ≥ 0.066). The Kruskal-Wallis sensitivity analysis yielded a consistent result (H(3) = 13.45, p = 0.004).

Dietary pattern associations with barriers and facilitators

Chi-square tests of independence were conducted to examine associations between the current dietary pattern and each barrier and facilitator item. Three barrier items yielded nominally significant associations that did not survive Benjamini-Hochberg correction across 12 simultaneous tests (false-discovery rate (FDR)-adjusted p-values noted below). Taste or texture of plant-based foods was associated with dietary pattern (χ²(6) = 18.46, p = 0.005, Cramér’s V = 0.356, FDR-adjusted p = 0.062); entirely plant-based respondents were most likely to report taste and texture as not a barrier (85.7%, n = 30), compared with 38.5% (n = 5) of omnivorous respondents. Knowledge for meal preparation was also associated with dietary pattern (χ²(6) = 14.82, p = 0.022, Cramér’s V = 0.319, FDR-adjusted p = 0.127); no plant-based respondents reported it as a significant barrier, compared with 38.5% (n = 5) of omnivorous respondents. Time for meal preparation showed a similar pattern (χ²(6) = 13.82, p = 0.032, Cramér’s V = 0.308, FDR-adjusted p = 0.127), with plant-based respondents least likely to report it as a significant barrier (14.3%, n = 5) compared with plant-forward (58.8%, n = 10), omnivorous (46.2%, n = 6), and vegetarian (50.0%, n = 4) respondents. Remaining barrier items (food availability when eating out, social pressure, cost, and conflicting nutritional information) did not yield significant associations (all p-values ≥ 0.163). No facilitator item was significantly associated with dietary pattern (all p-values ≥ 0.266). All chi-square analyses involved tables with expected cell frequencies below five; results should be interpreted with appropriate caution.

## Discussion

This study provides a cross-sectional psychosocial profile of adults attending an immersive WFPB lifestyle retreat, a population that has received little focused empirical attention despite the growth of intensive lifestyle medicine programming [16]. Three findings warrant discussion.

First, the markedly higher self-efficacy among groups further along the TTM continuum, with the pre-action group scoring markedly lower than those in action or maintenance (η² = 0.242), is consistent with the broader dietary behavior change literature. Ma et al. [17] documented a significant linear trend between TTM stage and self-efficacy for fruit and vegetable intake in young adults (p ≤ 0.05), and Henry et al. [18] found analogous stage-related differences in self-efficacy for healthy eating in difficult situations, including when away from home (p < 0.0001). While direct evidence for plant-based eating specifically is sparse [19], Vizcaino et al. [20] demonstrated that self-efficacy discriminates successful plant-based adherents from non-adherents (χ²(13) = 42.03, p < 0.001), with successful adherents reporting significantly higher confidence (median = 14 on a 16-point scale) than those still struggling to adhere (median = 11; p = 0.16).

The present findings extend this pattern to an older, highly motivated retreat-attending population and underscore that even volunteers seeking intensive intervention arrive with meaningfully heterogeneous self-efficacy profiles. The pre-action subgroup (n = 8) warrants particular attention: Kashani et al. [21] showed that early empowerment strategies can attenuate differences between low- and high-confidence participants. Additionally, Okpara et al. [22] identified ongoing accountability contact as a key driver of adherence, with participants eating differently in weeks they had staff contact versus weeks they did not. While Vizcaino et al. [20] note that action planning is more impactful for early implementation, and coping planning (the mental rehearsal of high-risk situations and pre-planned responses) is essential for behavior maintenance, strategic planning remains the strongest self-regulatory predictor of long-term dietary adherence (p < 0.001). Taken together, these findings highlight self-efficacy as a key target for early-stage participants in retreat programming.

Second, the barrier and facilitator profile reflects patterns documented in the broader literature, with important nuances. Food availability when eating out was the most commonly reported barrier (84.9%), consistent with Rickerby and Green’s [23] systematic review identifying accessibility as a dominant barrier in high-income populations. Henry et al. [18] and Okpara et al. [22] both note that restaurant and social eating contexts are precisely the settings where self-efficacy is most acutely tested, as these environments often provide limited options and require intentional substitution or navigation [18,22]. This convergence further supports the choice of social events and stress as the two self-efficacy assessment contexts in the present instrument. Dobson et al. [24] demonstrated that self-efficacy moderates the impact of stress on diet quality, with low-confidence individuals showing the steepest diet deterioration under stress, a pattern directly relevant to this sample’s pre-action members. The bimodal distribution of household support (rated very helpful and not a factor by equal proportions, 43.8% each) reflects the “support and sabotage” dynamic documented by Ortiz et al. [25] and Sriram et al. [26]: household influence on dietary change is highly variable, with some partners facilitating change through shared cooking and others undermining adherence through food-related pressure or indifference [25,26]. Post-retreat programming that helps participants anticipate and navigate their specific home environment may therefore be as important as the retreat itself, as sustained benefits often depend on follow-up coaching or community support [16,27]. These barrier and facilitator patterns underscore the importance of context-sensitive post-retreat support.

These findings should be interpreted in light of several limitations. The sample was predominantly White, female, older, and highly educated, limiting generalizability. The cross-sectional design precludes causal inference; more specifically, the concurrent measurement of barriers, facilitators, and stage of change means that the temporal relationship among these constructs cannot be established. It is not possible to determine whether perceived barriers and facilitators preceded, accompanied, or followed participants’ transition to their current stage, nor whether these perceptions shifted as they moved through stages. The absence of follow-up data means the relationship between pre-retreat psychosocial profiles and post-retreat behavioral outcomes remains untested. Health status and medical comorbidities were not assessed, precluding examination of whether clinical conditions influenced psychosocial readiness or self-efficacy; future program evaluations should capture this information. Additionally, because survey administration spanned the full retreat period, participants who completed the survey later may have under-reported barriers or over-reported facilitators relative to those who completed it earlier; this potential effect could not be disentangled within the present cross-sectional design. The two-item self-efficacy measure, while internally consistent (α = 0.718), has not been validated against established scales. Future work should explore whether motivation type moderates post-retreat outcomes in this population; notably, Vizcaino et al. [20] found that while general health motives (e.g., “to improve health”) may paradoxically predict a lower likelihood of adherence, those motivated by ethical beliefs or the management of a specific medical condition are significantly more likely to maintain long-term plant-based habits.

## Conclusions

In a voluntarily attending retreat population, self-efficacy for WFPB eating aligns with the TTM stage in a pattern consistent with the broader dietary behavior change literature. The preponderance of participants in later stages suggests that immersive programming attracts individuals who are meaningfully ready for change; however, a smaller pre-action subgroup with substantially lower self-efficacy represents a targetable intervention opportunity. Persistent environmental barriers, particularly eating away from home, point to the value of post-retreat support strategies that build coping self-efficacy for real-world settings. These findings may inform both program design and structured follow-up for intensive lifestyle interventions incorporating WFPB principles.
